# “I am a person but I am not a person”: experiences of women living with obstetric fistula in the central region of Malawi

**DOI:** 10.1186/s12884-017-1604-1

**Published:** 2017-12-21

**Authors:** Josephine Changole, Viva Combs Thorsen, Ursula Kafulafula

**Affiliations:** 1 University of Oslo, Department of Community Medicine and Global Health, P.O.Box 1130 Blindern, N-0318 Oslo, Norway; 2 University of Malawi, Kamuzu College of Nursing, Blantyre, Malawi

**Keywords:** Obstetric fistula, Lived experiences, Maternal health problems, Social consequences of fistula, Malawi

## Abstract

**Background:**

The consequences of living with obstetric fistula are multifaceted and very devastating for women, especially those living in poor resource settings. Due to uncontrollable leakages of urine and/or feces, the condition leaves women with peeling of skin on their private parts, and the wetness and smell subject them to stigmatization, ridicule, shame and social isolation. We sought to gain a deeper understanding of lived experiences of women with obstetric fistula in Malawi, in order to recommend interventions that would both prevent new cases of obstetric fistula as well as improve the quality of life for those already affected.

**Methods:**

We conducted semi-structured interviews with 25 women with obstetric fistula at Bwaila Fistula Care Center in Lilongwe and in its surrounding districts. We interviewed twenty women at Bwaila Fistula Care Center; five additional women were identified through snowball sampling and were interviewed in their homes. We also interviewed twenty family members. To analyze the data, we used thematic analysis. Data were categorized using Nvivo 10. Goffman’s theory of stigma was used to inform the data analysis.

**Results:**

All the women in this study were living a socially restricted and disrupted life due to a fear of involuntary disclosure and embarrassment. Therefore, “anticipated” as opposed to “enacted” stigma was especially prevalent among the participants. Many lost their positive self-image due to incontinence and smell. As a way to avoid shame and embarrassment, these women avoided public gatherings; such as markets, church, funerals and weddings, thus losing part of their social identity. Participants had limited knowledge about their condition.

**Conclusion:**

The anticipation of stigma by women in this study consequently limited their social lives. This fear of stigma might have arisen from previous knowledge of social norms concerning bowel and bladder control, which do not take into account an illness like obstetric fistula. This misconception might have also arisen from lack of knowledge about causes of the condition itself. There is need therefore to create awareness and educate women and their communities about the causes of obstetric fistula, its prevention and treatment, which may help to prevent fistula as well as reduce all dimensions of stigma, and consequently increase dignity and quality of life for these women.

## Background

Currently there are over 2 million girls and women suffering from obstetric fistula in Sub-Saharan Africa and South Asia [1]. Obstetric fistula is an abnormal opening between the vagina and the bladder (VVF) and/or rectum (RVF) resulting in continuous and unremitting urinary or fecal incontinence [2]. This condition is caused by prolonged obstructed labor which has not been treated in time, commonly by caesarean section [2]. Obstetric fistula is uncommon in industrialized countries, following universal access to safe delivery care and emergency obstetric care [3], rendering its preventability unquestionable. For example, Trovik and colleagues [4] reported an incidence of 16 per 100,000 live births over a period of 19 years, between 1995 and 2014, for Norway. The most common form of fistulas found in these high income countries are caused by injuries during surgery, cancer or radiation treatment [5]. However, in resource-poor settings, especially those in South Asia and Sub- Saharan Africa, obstetric fistula persists in large numbers; unsurprisingly due to poor access to emergency obstetric care services [1].

In a systematic review and meta-analysis on prevalence of obstetric fistula in South Asia and Sub-Saharan Africa, Adler and colleagues reported a prevalence of 1.2 per 1000 women of reproductive age (15–49) in India and 1.6 per 1000 women of reproductive age in Ethiopia in 2003 [6]. Prevalence of obstetric fistula varies between different African countries. For example, the Uganda Demographic Health Survey 2006, reported that 26 per 1000 women of reproductive age are suffering from this condition [7]. Biadgilign and colleagues in 2013 reported a prevalence of 10.6 per 1000 women who ever gave birth for Ethiopia [8].

Malawi is one of the poorest countries in Sub-Saharan Africa with the majority of its population living below the two dollars poverty line [9]. Approximately 80% of the population lives in rural areas [10]. Poverty, distance, and poor road infrastructures make it difficult for people to access health care services [11]. Although maternal mortality has decreased over the years from 1120 per 100,000 live births in 2000 to the 439 per 100,000 live births in 2016 [11], the rate is among the highest in Sub-Saharan Africa. Currently there is limited information on maternal morbidity in the country. However the high mortality rate indicates a high morbidity rate, based on the fact that every woman who dies from complications during pregnancy, twenty more suffer from acute or chronic morbidities, including fistula [12, 13].

Obstetric fistula has been one of the neglected conditions in Malawi, drawing attention only after the launch of the End Fistula Campaign by United Nations Population Fund (UNFPA) in 2003 [14]. Anecdotal reports state that approximately 20,000 women are currently living with obstetric fistula in Malawi (approximately 5 per 1000 women). The Malawi Demographic and Health survey (MDHS) in 2004, reported a prevalence of 1.6 per 1000 women of reproductive age (15–49), similarly the MDHS 2016, states that 1 % of women have reported experience of obstetric fistula between 2010 and 2015 [11, 15]. In a national survey conducted in 2007 on incontinence, a proxy measure of vaginal fistula, Johnson [16] reported a lifetime prevalence of obstetric fistula of 15.6 per 1000 women of reproductive age. And in yet another national community survey on prevalence of fistula in 2010, Kalirani and colleagues, reported a lifetime prevalence of 1.6 per 1000 women [15]. Due to varying methods and reporting from these surveys, the real prevalence of the condition is not known.

Studies on prevalence of fistula mainly rely on patients’ self-reports or siblings’ reports about incontinence observed following childbirth. Such self-reports may be overestimating the real prevalence of obstetric fistula by including urine and/or fecal incontinences caused by other conditions. On the other hand, women may choose not to report their condition due to fear of stigma, leading to underestimation [8, 17]. Lack of reliable data about obstetric fistula in Malawi may have an impact on policy decisions concerning resource allocation for an already neglected condition like fistula.

Obstetric fistula is treatable with 80–98% closure rate, with proper surgical technique and adequate resources [18, 19]. This implies that most women could potentially regain their continence with just one attempt of fistula repair. Unfortunately, due to shortage of skilled surgeons and resources, women continue to live with the consequences of fistula for a long time; and in some cases for the rest of their lives [2, 8, 20].

The impact of obstetric fistula is multifaceted, having both medical and psychosocial consequences. Having urinary and fecal incontinence makes it difficult for individuals to maintain good hygiene and carry on ordinary social and working lives [21]. The incontinence causes deep ulcerations on the woman’s external genitalia, which may extend to the buttocks and thighs, resulting in pain and difficulties in movement. Additionally, the loss of the fetus that normally occurs due to prolonged and untreated obstructed labor could reduce the woman’s prospects of a better marital relationship; especially in societies where childbearing carries strong social expectations. Lack of awareness and knowledge about the cause and treatment of fistula by family and community members, may lead to misconceptions that may further subject these women to ostracism and stigmatization, making their general quality of life miserable and unbearable [2, 6, 13, 17, 22, 23].

Stigmatization is one of the worst experiences one can have on top of a devastating condition like fistula. Goffman describes stigmatization as “the process of devaluation within a particular culture or setting where certain attributes are seized upon and defined as discreditable or unworthy” [24]; in this case it could be the leakage of urine/feces and the smell thereof. Over the past two decades researchers such as Stuenkel and Wong [25], Quinn [26], Chaudoir and colleagues [27], and Link and colleagues [28] have modified and refined the work of Goffman. Consequences of stigmatization may include self-devaluation, self-isolation, depression, reduced life chances, suicidal thoughts and impairment in social involvement. Furthermore, stigmatization acts as a barrier to seeking help and accessing support from others [26, 27].

Although stigmatization is a commonly reported social consequence among women with obstetric fistula, there is limited knowledge on how it is specifically experienced by women with obstetric fistula. Furthermore, there is limited knowledge on women’s, families’ or communities’ understanding of the causes of obstetric fistula. If communities and the women themselves are misinformed or have distorted views about the origins of the condition, there is a risk of social misconceptions and stigmatization [22]. Having a deeper understanding of these consequences based on lived experiences from a Malawian context will contribute to the design of effective interventions [29]. In this study, we sought to get a deeper understanding of how women experience living with obstetric fistula in Malawi, in order to make recommendations to improve the quality of life for these women as well as help them to re-integrate into their communities.

### Theoretical framework

The study drew upon the concepts of stigmatization. As mentioned earlier, stigmatization is the process of devaluation within a particular culture or setting where certain attributes are seized upon and defined as discreditable or unworthy [24]**.** Stuenkel and Wong [25] state that stigma arises from widely held social norms and beliefs about personality, behavior and illness and is communicated through a process of socialization. The process of stigmatization may be described as follows: “the society teaches its members to categorize persons by common defining attributes and characteristics. The society also labels and adopts other behaviors or characteristics as not conforming to norms and as discrediting" [25]. Therefore, when a person displays behaviors or characteristics that are marked as discrediting, such as incontinence of urine/feces and smell, she may be set apart, labeled as different or deviant. Now we ‟the so called normal” tend to identify the person by the discrediting attribute; for example ‘the incontinent’ or the ‘stench’ (or we may even use a derogatory term to label them as such) disregarding all other important attributes that she may possess. The label (stigma) that she now acquires leads to social devaluation, exclusion from social interactions, altered social relationships, status loss and discrimination. Discrimination may occur at both individual and structural levels. At individual level, the stigmatized person may experience name calling, gossip, violence and rejection from peers. At structural level, there could be regulations or policies that work to disadvantage a stigmatized group of people or person; for example, denied employment or access to an organization. In response to the stigmatization, the person may end up stigmatizing herself. Stigma can take many forms: anticipated, enacted, internalized and associative stigma [24, 25].

*Anticipated (Felt) stigma* refers to the degree of fear, which individuals expect that others will stigmatize them if they know about the discrediting attribute or characteristic. Discreditable characteristics or attributes are ones that can be hidden from others and that are socially devalued and negatively stereotyped [26].

*Internalized (self) stigma is* where the stigmatized individual tends to hold the same beliefs about the identity that others have of her. This attitude or feeling towards oneself may lead to self-discrimination, for example, one may stop herself from participating in an activity because she feels useless [26]. Internalized stigma incorporates feelings of shame, dejection, self-doubt and sometimes feelings of guilt, self-blame and inferiority.

*Enacted stigma* refers to the real experience of discrimination because of the stigmatizing condition [26]. This can be manifested through blaming, discrediting, gossip, verbal harassment, avoiding contact, ostracism and abandonment [30].

Both internalized and enacted stigmas can lead to anticipated stigma.

An *associative stigma* (also known as a courtesy stigma or affiliate stigma) is a stigma that a person possesses because of his or her close connection to a stigmatized other [24, 26]. For example, a family being shunned because one of its members has a stigmatizing condition. In the following section we present the methods used in this study.

## Methods

### Study design and setting

The qualitative research was carried out between June 2015 and September 2016, at Bwaila Fistula Care Center in Lilongwe. Bwaila Fistula Care Center is primarily dedicated to treatment and repair of genital fistulas; most of which happen to be obstetric fistulas. The Center has 35 beds, which cater to both preoperative and postoperative fistula patients. It was established by Freedom from Fistula Foundation in 2010, and was officially opened in 2012. Since its establishment in 2010, approximately 400 women with different kinds of fistulas are treated every year [31].

### Study participants.

Twenty-eight women diagnosed with obstetric fistula were eligible to participate in this study. Three of them refused to participate, making the final number of participants 25. Twenty of the women were purposively [32, 33] selected from Bwaila Fistula Care Center, while five were recruited through snowball sampling [32, 33], through women who had been recruited at Bwaila Fistula Care Center. The women recruited through snowball sampling were visited in their homes and requested to participate in the study. They were women who had previously been diagnosed with fistula, but had not yet sought treatment at the Fistula Care Center. To be eligible, the woman had to be: diagnosed with either vesico- vaginal fistula (VVF) or recto-vaginal fistula (RVF) or both, admitted at Bwaila Fistula Care Center, waiting for surgery or recovering from surgery, living within the Center’s catchment area and within the Central Region, regardless of their age and duration of living with fistula, and willing to participate in the study. Twenty family members also participated in the study. The participant herself decided on which family member to be interviewed. They included people who knew about the woman’s condition and supported her to cope with it.

### Data collection

Semi-structured interviews [32, 33], which lasted between 30 and 90 min, were conducted by the first author. Questions focused on demographic information, historical accounts of pregnancy that led to fistula development and experiences of living with fistula. To increase trustworthiness [34], the first author conducted follow-up interviews with 10 participants to confirm issues arising from participants’ earlier interviews.

Semi-structured interviews were also conducted with participants’ family members to gain their views and perspectives about fistula and to triangulate data from the participants. These latter interviews were conducted in the participants’ homes. Triangulation [34] helped strengthen the trustworthiness of the data by providing further insights on what their loved ones shared. Questions focused on: awareness of fistula before their relative developed it, understanding of the cause, how other people reacted and treated their loved one and their views on economic effects of fistula. Interviews lasted between 15 and 30 min. To minimize researcher bias, the researcher kept a journal of personal reflections about the data collection process and continuously reviewed findings with [34] the second and third author throughout the data collection period.

### Data analysis.

Analysis of the interviews was done concurrently with data collection process. All the interviews were transcribed verbatim and translated from Chichewa to English by the first author and three research assistants. Three transcripts were given to a linguistic teacher to back translate to Chichewa to ensure meanings had not been lost in translation; and there were no significant differences. The first author did the final editing by checking all the transcripts against audio recordings to ensure accurate transcription and translation. A transcript was further checked against an audio recording whenever something did not make sense while reading it. The English transcripts were used for analysis and the original transcripts were crosschecked to ensure a correct interpretation throughout the process. The process was guided by a thematic analysis approach that was both deductive and inductive in nature [35]. Deductively, previous studies and literature provided information on how some women have been treated in other countries. The pathway to obstetric fistula identified the causes and social consequences, which included stigmatization and thus required a working knowledge of the theoretical framework of the dimensions of stigma. In other words, the data were reviewed/analyzed with certain preconceived categories derived from the aforementioned theory and conceptual framework while allowing for themes to emerge directly from the data using inductive coding. Inductively, the transcripts were carefully read sentence by sentence to obtain a sense of the content as narrated by participants and compared to field notes.

Phrases and sentences related to experiences of obstetric fistula were coded in the margin of the transcript sheets, and codes with similar content were combined into categories. Nvivo 10 software was used for data management. Several rounds of discussions among the co-authors were necessary to strengthen credibility and integrity of the findings [34]. To ensure confirmability, all the co-authors reflected, discussed differences in interpretation of data and agreed on the categorization [34, 36].

### Ethical consideration

The study was conducted in compliance with the principles of the Declaration of Helsinki [37]. Ethical clearance was obtained from the College of Medicine Research Ethics Committee (COMREC); reference number **P.03/15/1711.** The study protocol was exempted by the Regional Ethics Committee (REK) in Norway and was registered with the Norwegian Center for Research in Norway; project reference number **2014/2040/REK.** All participants gave both oral and written informed consent after discussing the purpose of the study and issues of confidentiality. All transcripts were anonymized and pseudonyms are used in this report to protect participants’ identities and to ensure confidentiality. Voluntary participation was emphasized. Women were given an equivalent of $2 for transport to the hospital for those recruited through snow ball sampling or back to their homes if recruited at the care center. This was done at the end of the interview to avoid undue inducement [37].

## Findings.

### Participants’ characteristics.

A total of 28 women diagnosed with obstetric fistula were invited to participate in the study. Three women refused to participate. Of the 25 women who participated, six did not know their age. Of those who did, their ages ranged from 16 to 67 years. Years of education ranged from 0 to 11 years, with the majority having no formal education (*n* = 14). The majority were still married at the time of the study (n = 14), 5 were divorced, 4 were widows and 2 were single. Almost all were subsistent farmers. Their characteristics are presented in Table 1. Twenty family members participated, including sisters, mothers, mothers-in-laws, husbands, a brother and aunts. Their characteristics are presented in Table 2.

**Table 1:** Characteristics of participating women with fistula

ID	Age group	Years in school (range)	Parity	Children alive	Pregnancy causing fistula	ANC at least attended once	Type of delivery	# of days in labor	Fetal outcome	Years/month(m) with fistula
1	33–42	0–3	3	2–3	2	Yes	CS	3	SB	12
2	33–42	4–7	5	2–3	3	Yes	Normal	2	SB	12
3	N/A	0–3	7	4–5	7	Yes	CS	1	SB	10
4	16–32	8–12	2	2–3	2	Yes	Normal	1	Alive	1
5	16–32	4–7	1	0–1	1	Yes	CS	5	SB	1
6	16–32	8–12	1	0–1	1	Yes	CS	1	SB	5 m
7	16–32	0–3	1	0–1	1	Yes	Normal	1	SB	4 m
8	16–32	4–7	4	2–3	4	Yes	CS	1	SB	4 m
9	16–32	0–3	6	2–3	1	Yes	CS	2	SB	16
10	N/A	0–3	5	0–1	5	Yes	DVD	1	SB	15
11	43–67	0–3	6	2–3	6	Yes	CS	1	SB	19
12	N/A	0–3	6	4–5	6	No	CS	2	SB	4
13	33–42	4–7	2	0–1	1	Yes	Normal	3	SB	15
14	33–42	0–3	2	0–1	2	No	CS	2	SB	20
15	16–32	8–12	4	2–3	4	Yes	Normal	2	SB	1 m
16	33–42	0–3	2	0–1	2	Yes	CS	0	SB	19
17	43–67	0–3	2	0–1	2	No	CS	1	SB	21
18	33–42	0–3	8	2–3	1	No	Normal	1	SB	19
19	N/A	0–3	7	2–3	6	No	Normal	2	SB	DK
20	43–67	0–3	3	0–1	3	No	CS	1	SB	45
21	33–42	4–7	1	0–1	1	No	Normal	2	SB	12
22	N/A	0–3	5	0–1	5	Yes	Normal	1	SB	13
23	16–32	0–3	5	2–3	5	Yes	CS	1	Alive	3 m
24	43–67	0–3	2	0–1	2	Yes	CS	2	SB	27
25	N/A	0–3	13	0–1	1	Yes	Normal	1	SB	47

Abbreviations: N/A = age group not applicable because the participant did not know her age, CS = Caesarean section, Normal = vaginal delivery, DVD = destructive vaginal delivery, SB = stillbirth, DK = doesn’t know/ remember

**Table 2:** Characteristics of family member participants

ID	Age group	Sex	Years in School (range)	Relationship to participant
1	N/A	1	0–3	Mother
2	34–42	1	4–7	Sister
3	N/A	1	0–3	Cousin
4	52–60	1	0–3	Mother-in-law
5	34–42	1	0–3	Sister
6	34–42	1	8–12	Aunt
7	43–51	1	0–3	Daughter
8	34–42	0	0–3	Brother
9	25–33	1	8–12	Sister
10	N/A	1	0–3	Mother
11	25–33	0	8–12	Husband
12	N/A	1	0–3	Aunt
13	52–60	0	4–7	Husband
14	52–60	0	8–12	Husband
15	N/A	1	0–3	Mother
16	34–42	1	4–7	Sister
17	43–51	1	0–3	Sister
18	39	0	4–7	Husband
19	51	1	0–3	Mother-in-law
20	N/A	1	0–3	Sister

Abbreviations: N/A = age group not applicable because the participant did not know her /his age, 0 = Male, 1 = Female,

In the following sections we present three themes identified in the study: 1) awareness, knowledge and perceptions about fistula, 2) barriers to seeking healthcare, 3) experiences of stigmatization with the following sub-themes: experiences and consequences of anticipated (felt) stigma, experiences and consequences of internalized stigma, experiences and consequences of enacted stigma, and experiences and consequences of associative stigma.

### Knowledge, awareness, and perceptions about fistula

#### Awareness of fistula before diagnosis

In order to learn about the awareness, knowledge and perceptions about the causes of fistula, participants were asked whether they had ever heard of or seen any woman with leaking urine or feces in their community, and what they thought had caused that individual’s condition prior to their own diagnosis. The findings revealed that participants had little prior awareness and knowledge about their condition. The majority of participants (*n* = 16) stated that they had never heard or seen any person with the condition before. They further indicated that they found out about the condition when they had developed it, thus, to their knowledge, they were the first sufferers in their communities. This fact left participants with mixed reactions; some were worried, dismayed, confused or afraid, as illustrated by the following quotes:

*“I was so worried because I had never suffered from this problem before. So I was afraid that, "what kind of life will I be living with a condition like this?"* ” **[Nadimba, 1 month living with fistula]**

Another participant said:

*“This operation was not the first one, but a third one. So I was surprised during this operation, that “ah, all these times I have had operations, were I wetting myself or my bed? What is happening now?” At first I thought, “Maybe it is because of the wound since it is still fresh, when the wound heals, the urine will stop”. But I saw that no, the wound had healed but the urine is not stopping.”* **[Nabengo, 4 months living with fistula]**

Some participants had heard about the condition through friends and/or radio, while others had previously seen women in their communities who had just been getting wet, but could not tell what had caused them to be in that state.

In two cases it was discovered that participants had parents who had also suffered and been treated for obstetric fistula. As described by participants in the following quotes:

*“She [my mother] told me that, “it is fistula, you will be cured, I was also doing like this [leaking urine]”. So I was not worried much… because she has also been to this hospital before. …she had it [fistula] from 2007 to 2014.*” **[Nambewe, 1 year living with fistula]**

Another participant also said:

*"When I explained to my mother she said, “The way you are explaining your problem is the same way I experienced it when I gave birth to your brother, the last born. …what it means is that your bladder was worn out on one side. And your breaks that stop urine are loose, that is why you are failing to keep your urine… As it is, you must be putting on cloth pads in order to trap the urine, maybe, that would help to slow down the leaking little by little. Yes, that is what she told me.”* **[Nanyoni, 12 years living with fistula]**

These two reports were later confirmed through interviews with their family members without necessarily disclosing what the participants had revealed in their interviews. When asked whether she knew or had seen any woman with a condition of leaking urine before her daughter had developed it, this mother of a participant responded like this:

“*A problem like this [leaking urine], hearing about it, I have heard, but at the same time, I myself, have suffered from fistula, but it was of urine only. And only to see that my own child has gone to give birth and this [fistula] has also happened to her. Iii, it touched me so much, and it happened at Bwaila [referring to a hospital]*. *So I just said, let us go back to hospital. Really.”* **[Mother to Nambewe, lived with fistula for 7 years before her daughter got it, now repaired]**

#### Perceived causes of fistula

The study revealed that participants had very limited knowledge about the causes of their condition. Although most of them could associate the onset of their condition with childbirth, they could not understand or explain what might have happened during labor or delivery to bring about the condition. Therefore, they came up with different ways to explain the causes of their condition. Only three participants related the condition to delay in delivery of their babies. As one explained it like this:

*“I just thought that it may be because the baby had stayed too long in my womb. Because the baby really went through a lot, yes, went through a lot of problems, struggling to come out. He could not breathe; he was stressed a lot when he was in there. So because of this, I thought maybe due to the stress, he had torn something open in there [her womb]. Indeed.”* **[Namauya, 19 years living with fistula]**

Over half of the participants delivered via caesarean section as a way to alleviate the obstructed labor. As such, they attributed the development of their condition to the operation and errors made by the surgeons during the caesarean section. As conveyed by the participant in the quote below:

*“In my mind I thought my bladder had been cut during the operation. Is it not the bladder that keeps urine? So in my thoughts, I thought they did not do the operation well, as such, they had cut my bladder a little.”* **[Nasibeko, 16 years living with fistula]**

And another participant sharing the same sentiments said:

*“I was thinking that I was injured when they were operating on me, that maybe they made a mistake somewhere. That was what I was thinking.”* [**Namoyo, 1 year living with fistula]**

Some participants in the study had no idea at all of what might have occurred for them to develop the condition. As such, some attributed it to witchcraft, and so did some family members. One participant believed it was the work of the devil. One participant attributed it to unfaithfulness of her husband and so did one family member.

Blaming her husband for her condition, this participant stated it like this:

“*What pained me is that he would leave me at home while heavily pregnant, meantime he would sleep out; three days, four days. When he comes back, we have sex. “Do I know where he slept?” No. That is why I say that it could be the marriage that caused this problem…they say that if a man sleeps with another woman and then sleeps with his pregnant wife, there is a mix of bad blood and this problem can happen.”* **[Nasiketi, 17 years living with fistula]**

And this family member also suggesting that the husband’s unfaithfulness had something to do with the development of fistula said:

*“Some say she has been bewitched; I don’t know. Or the man was sleeping with other women. You see, previously people were taking care of the pregnancy, you, her mother was taking care of it too. I was just asking myself “is it the man? I don’t know”. “Could it be that the man was sleeping around and has made the thing [the unborn baby] to be stuck and to tear the person [her niece] apart?*” *Really.*” **[An aunt to Naphiri, who has lived 15 years with fistula]**

And blaming witchcraft for her condition the participant in the quote below said:

*“I thought it was created for me. …I mean that, maybe someone used magic on me to be leaking water*”*.* **[Nachisale, 45 years living with fistula]**

And another participant blaming the devil for her condition she described it like this:

“*My belief is that the devil has had a hand in this, for me to have this problem. The devil really had his hand in this without a doubt. Because all my children died and now I have gotten injured. When I look around, my God is merciful, and because He is merciful, He could have forgiven me for at least one thing; so it is not Him. But the devil really had his hand in this.*” **[Nabiyeni, 15 years living with fistula]**

### Barriers to seeking health care

Lack of access to health services for women with obstetric fistula subjected most of the participants to prolonged suffering with the condition. The study revealed that all participants sought some kind of help for their condition at one point or another. Because most of the participants developed fistula before the Fistula Care Center was established in 2010, the majority of them first sought treatment from public district hospitals; however, services for fistula repair were not available in most of these hospitals. Participants had to be referred to a tertiary public hospital, which, to their disappointment, also did not have skilled doctors to attend to their condition. Participants reported having had to visit the hospital on several occasions and being turned back, because the doctor was not available. In some cases no justification was given for refusing to admit the patient, as suggested by the participant in the quote below:

*“But mmm, people at this place [hospital] oppressed me so much, mmm. For them to help me, no. The doctors here said, “Come on 16 March”. I came and they said, "Come another day. I came on that day, and they told me come on another day, I came and was told to come another day again. That was when I decided to proceed to Nkhoma (a Private Mission Hospital).”* **[Nachisale, 45 years living with fistula]**

And reporting on unavailability of doctors one participant described her dilemma like this:

“…*because at that time there was only one doctor called.... He was coming from outside the country just to help people with this problem [fistula]. So we were thinking that, “even if we go back there again, will we find the doctor?” Because the first time we went there [Central hospital], we were told that the doctor was outside the country and we had been sent back.”* **[Nadzimbiri**, **15 years living with fistula]**

Some participants, having not been helped at the public tertiary hospitals, continued to look for help from other sources such as private hospitals, mission hospitals and traditional healers. But they, too, eventually gave up. Two participants sought help from private hospitals and ended up being misdiagnosed, mismanaged, and given false hopes by health workers as narrated by the participant in the quote below:

*“At the private hospital I was given drugs for a sexually transmitted disease, because I was told, “maybe it could be a sexually transmitted disease, so we will give you an injection and drugs to take together with your husband. So, me and my husband we would both go for the injection and we would take the drugs together, and they said, “don’t have sex while you are on this treatment so that the disease should end.”But I saw that the problem never ended. During all this time my husband and I never stopped thinking of ways to solve my problem.””* **[Nasibeko, 16 years living with fistula]**

Participants reported several reasons for their giving up on seeking medical care, including lack of transport, family responsibilities, lack of financial support, long distances to the health facilities and lack of knowledge on available treatment for the condition.

### Experiences and consequences of stigmatization

During the period of living with urine and/or fecal incontinence, participants experienced varied forms of stigmatization, which manifested in the form of shame, social and self- isolation, low self-esteem, ridicule, abandonment, feelings of worthlessness, and self-devaluation. In a few cases, relatives to these participants also fell victim to stigmatization due to association with the participant. The varied forms of experienced stigmatization are presented in the following sections.

### Experience and consequences of anticipated stigma

#### Fear of involuntary disclosure and embarrassment

All participants in the study had a fear that others would humiliate, embarrass or isolate them should they discover their condition (anticipated stigma). They apparently developed this fear even without any previous negative experiences due to the abnormality of their bodily functions (i.e. uncontrollable leaking of urine and/or feces) and the offensive smell. They, therefore, worked tirelessly to hide their condition from others. They changed and totally adapted to new routines in order to conceal their condition. Frequent washing and changing of cloth pads as well as frequent bathing became part of their daily routines. They also limited or completely avoided social gatherings such as church, wedding ceremonies, funeral ceremonies, markets and any other public places, which in their mind could potentially lead to involuntary disclosure should they find themselves leaking as suggested by the participant in the quote below:

*“I could not manage to stay in a group; I have to be honest. Because it can happen that I am putting on a cloth pad, and if it is not thick enough, it means I have exposed myself; everyone will laugh at me. Not due to smell, no; I will be lying if I say so. But due to the cloth getting wet or if it was in contact with my skirt, then it would mean that the skirt is also wet. So people would be saying, “look, look”, so I find that to be a burden.*” **[Nasiketi, 17 years living with fistula]**

And portraying similar sentiments another participant said:

*“I was feeling embarrassed that, “when I go there [to church] and I want to stand, what stories will I leave behind?” Because you may put on the cloth pad and go to church, and during the preaching you may find yourself wet, what are you going to do? Or you may want to stand, and you find that you have soaked the place where you were sitting, what are you going to do? You will be embarrassed, because people will now know that, “ah, is this person like this?” So it will be like you are going to expose yourself that you have such a disease. So, I just stay at home, that, “if I have to be embarrassed, then, let it happen here at home.*”” [**Nabengo, 4 months living with fistula]**

Two of the seven divorced participants remarried with their new husband’s full knowledge of their condition; however, other participants deliberately chose not to remarry due to fear of disclosure, which could lead to abandonment.

One participant describes her dilemma in the quote below:

*“Different men proposed to me for marriage, saying “woman I love you I want to marry you and I know that you are divorced”, I would tell them, “I don’t want to get married. I was married before, but I don’t want to get married again now, it is not yet time to get married again.” I did not reveal the real reason to them. But I knew that, the ones who are getting married know that they are dry, everything is okay. But as for me, that one [her ex-husband] has seen me, he got drunk and exposed me, I get married to another one, he would expose me again for the second time, then another, will I survive? No. As it is now, I left all my worries; I don’t even have sexual desire. When women talk about marriage, I just listen to them.*” **[Nadzimbiri, 12 years living with fistula]**

### Experience and consequences of internalized stigma

#### Loss of self-value/ feeling of worthlessness

Some participants developed negative self perceptions due to negative reactions from friends following an involuntary disclosure of their condition or due to the knowledge about other women who experienced insults, devaluation or discrimination due to symptoms similar to their own. This led to, self-devaluation, loss of confidence and self-esteem.

The participant in the quote below conveyed her feelings like this:

*“My life is different now,… as it is now, I am a person but I am not a person, because sometimes, feces just come out without control. …a real person is orderly. For example, as you are there, you are walking and suddenly you find yourself with feces, would people take you for a real person? No, they can’t take you for real, they would say “this woman is crazy” that is what I mean; yes. So, you look at a person, she looks okay, but what she does; abnormal.* **” [Nankhoma, 1 year living with fistula]**

And another participant sharing similar feelings also said:

*“I am always wet on my bottom; even my half petticoat is so stained and worn-out at the back. When you try to wash, the water becomes so dirty; it is so pathetic.” And I think to myself, “Oh my God, what happened to me?” My friends are all fine. “Ah! (sighing), I was a person previously, what has now happened to me?”* **[Nachanza, 15 years living with fistula]**

### Experience and consequences of enacted stigma

#### Abandonment by spouse

While the majority of the participants remained married, seven participants were abandoned by their husbands when it was discovered that the condition was becoming chronic and was disrupting their sexual relationship as reported by the participant in the following quote:

“*He divorced me because of this problem [her leaking], he divorced me because of this problem [sounding so sad]”…he was saying, “my wife you are lame, I am not going to fish”… fishing means sleeping together as husband and wife [having sex]. So he said, “I will not fish like I did before, it is better that we separate.*” **[Nalike, 4 years living with fistula]**

Conversely, for participants who remained married, it was reported that their husbands accepted them with the condition as they saw themselves as being partially or totally responsible for the development of the condition as suggested by the participant in the quote below:

*“He used to tell me that don’t be sad, I cannot forsake you. This problem has come because of me. If you were not pregnant, this problem would not have been there”. … There were other people who were telling him many things like, “Why are you keeping a disabled woman? Why can’t you marry another woman?” So he used to tell them that, “don’t make me sin, I found this person in good condition when she was at her home. No one becomes pregnant without someone, so if I accepted the situation, no one can make a decision for me.*” **[Namasina, 19 years living with fistula]**

Complementing the above sentiments another participant who was now widowed at the time of the study but had lived with her husband for 15 years while with fistula stated it like this:

*“My husband persevered. Many people told him, “How are you going to live with your wife, who is disabled like this?” He would tell them, “When I came to propose to this woman, she was very well; this problem came with me. Because if I did not make her pregnant, she would not get damaged as she is now, but it is because of me. So you tell me to leave her? No, I cannot leave her, God will free us”. So we stayed together until the time he died.”* **[Nashawa, 36 years living with fistula]**

#### Verbal abuse

There were few reports of verbal abuse among the participants. Only two participants reported being avoided and verbally insulted by their relatives. This participant complained like this:

*“I hear them (her siblings) talking about me, concerning my behavior, “Look at her, why does she behave this way? Is she alright in the head? This one has a problem. Go away from there, water pool”. I cry a lot, feeling sorry for myself, wondering what is wrong with me.”* **[Namauya, 19 years living with fistula]**

### Experience and consequences of associative stigma

#### Insult to injury

There was very low associative stigma reported in our study. Besides the experiences described by women in above sections, where men remained with their wives but had heard negative comments about their marital life, only one man of the twenty family members interviewed reported being insulted and disrespected because of his wife’s fistula as illustrated in the quote below:

*“Mm, it was a burden. ... Even us, their husbands, we do not have real peace. For you to talk about hygiene to other people it is so difficult. They say, “Uh! Look at the chief, he is talking about the hygiene of our tearooms, yet he stays with damaged things”. So it really brings mockery and insults on someone.”* [**Chief, and husband to Nalisewo, 7 years living with fistula**]

The above case of associative stigma led the man to marry another wife and spend much of his time with the second wife as noted by Nalisewo, the first wife who was interviewed earlier.

## Discussion

In the current study we sought to understand how women experienced living with obstetric fistula. The testimonies of the women in our study illustrate similarities and variations in their experience. The women had limited knowledge about the cause of their condition; they experienced barriers to access treatment and all forms of stigmatization. The findings indicate that women experienced more of anticipated stigmatization as compared to the other forms. Women had fear of being discovered and hence being humiliated or embarrassed, so they responded by self-isolation, avoidance of social gatherings and public places and self-devaluation. These findings are similar to findings from previous studies [23, 38]. It may be argued here that the anticipation of stigma demonstrated by women in our study is not surprising, considering that stigma arises from widely held social norms about personality, behavior and illness, communicated to individuals through the process of socialization [24]. Furthermore, substances like feces, urine, vomitus and other bodily fluids are considered disgusting and unhygienic in most cultures and hence call for privacy and proper management in terms of when, where and how to dispose of them, in order to maintain proper hygiene [35]. Hence one learns the art of managing such bodily substances in a socially acceptable manner at an early stage in life. For example, in most cultures one is socially expected to acquire and maintain bladder and bowel control at as early as five years; and that socially signifies self-reliance [39, 40]. Losing control over one’s bladder and/or bowel movement might therefore be contrary to social expectations, hence attract ridicule, humiliation, embarrassment, anxiety, shame, loss of dignity and social isolation [35, 40]. The women in our study might have reflected on such socially grounded norms to anticipate negative reactions from others. Therefore, to avoid such reactions, they responded by concealing their condition from others; disclosing to close relatives only, who in turn kept the condition secret. But while this arrangement might have helped the women and their families to avoid stigmatization, such behavior has negative health implications. Lack of disclosure is associated with lack of support, poor health seeking behavior and poor compliance to treatment; and, consequently, leading to poor disease management [27]. The women in our study also experienced some enacted stigma in a form of abandonment by spouses. The main reason for this type of stigma was sexual dissatisfaction due to disruptiveness of urine or feces during sexual intercourse. This finding is similar to a study on experiences of women living with fistula done in the southern region of Malawi, where they found high numbers of divorce and remarrying among women due to fistula [23]. Contrary to the above study, only five women were divorced at the time of our study and only two had ever been divorced and remarried. Worth noting is the fact that the majority of the women were still married and living with their husbands. This finding is similar to a study done in Uganda on experiences of men living with wives suffering from obstetric fistula, where they found that all the men who participated in the study continued their marriage. The reasons given for their choosing to continue in the marriage with a woman suffering from obstetric fistula included love for their wives, marriage being a norm that includes responsibility for a wife and children, the feeling that they shared in the process that led to fistula and lack of money to marry another wife [41]. Apart from what women reported about their husbands’ blaming themselves for playing a role in the development of fistula, it was beyond our study to learn more on why the husbands chose to continue their marriages, because very few participants suggested a husband as a family member to be interviewed. Compared to reported findings in a previous study [42], our participants reported few instances of verbal abuse. This could be explained by the women’s ability to hide their condition or to isolate themselves from others.

Compared to reported findings in a previous study on experiences of family caregivers in Ghana [43], only one man of the twenty family members interviewed in our study reported associative stigmatization. Although this was the case, its existence needs consideration. Associative stigma may lead to negative responses against the individual who is the source of the stigma. Goffman [24] suggests that as a response to associative stigma, which he calls “courtesy” stigma, an individual may react by avoiding or terminating the relationship altogether. In our case, the family member in question reacted by marrying a second wife and spending as little time as possible with our participant, thereby denying her the most wanted and needed support in her life. What also is of concern in this case is that the person who experienced the associative stigma was a community leader. The importance of community leaders in health issues need not be overemphasized, considering that they are a group of people who are trusted and respected by their communities, hence influential. For example, in a community participatory intervention to reduce HIV-related stigma in Viet Nam, community leaders were trained to raise awareness and change community norms and reduce fear of HIV; consequently reducing value- and fear-driven stigma [44]. The experience of associative stigma in our study and the consequence of a community leader disassociating from his wife, expose a barrier to such leaders carrying positive messages about the condition and reducing stigmatization. Just like the other forms of stigma, creating awareness and educating the public about the causes, prevention and treatment of obstetric fistula may help to create understanding and reduce this type of stigma.

Apart from stigmatization, findings from our study also indicated that there was generally poor awareness and understanding about obstetric fistula. The majority of women had no idea at all about what caused their condition and hence associated it with caesarean section and doctors’ surgical errors among other causes. Similar findings were reported in a study done in Nigeria, where despite the majority of women correctly identifying prolonged labor as the cause of fistula, some women attributed their condition to the very operation that helped them, and opted not to undergo an operation in their subsequent pregnancies [45]. While some fistulas may indeed develop as a result of poor surgical techniques [46], the link between obstetric fistula and caesarean section found in our study raises great concern; especially considering that caesarean section is the most appropriate intervention for obstructed labor if conducted in a timely manner [2, 20]. Relating the development of obstetric fistula to caesarean section may therefore undermine the strategies to save lives of mothers and babies and to reduce the occurrence of this condition. There is need therefore to give women correct information about causes and treatment of obstructed labor, the main cause of fistula, in order to refute the misperceptions about caesarean section. Health care workers need to be equipped with adequate knowledge about the cause, prevention and treatment of obstetric fistula through refresher courses, workshops or continuing professional development sessions, in order to improve awareness at antenatal, family planning, under five and out-patient clinics. They can also conduct public campaigns to create awareness and educate the public about fistula, its causes, prevention and treatment; curbing the stigmatization process in its tracks.

Findings from our study have also indicated that most women actively sought medical help for their condition as soon as they noticed the symptoms. However, their efforts to seek out treatment were in vain due to unavailability of doctors to do surgical repairs. These findings are contrary to what was reported in studies done in Ethiopia and Nigeria where they found that women first sought help from different places before seeking medical care for their condition. Reasons given in the two studies included lack of knowledge about cure and cultural and religious beliefs [22, 47]. However our findings are in line with findings from a UNFPA study on obstetric fistula needs assessment in nine countries, in which Malawi participated [20]. In their study the authors highlighted lack of skilled surgeons as one of the major challenges in the management of obstetric fistula. Specific to Malawi, the authors also reported high workloads of complicated cases and limited theatre space, rendering fistula repair of less priority [20]. This might have been the case, because the study in question predated the current Fistula Care Center. This might also explain why most women who developed fistula before the establishment of the Fistula Care Center lived with the condition for many years without repair. This might point to a larger issue regarding training of midwives. There is a critical need for monitoring the effective management of women in labor to be reemphasized and repeated throughout the training so that appropriate emergency obstetric care is provided and obstetric fistula prevented.

Apart from facility based barriers, women also reported lack of transport and lack of knowledge about availability of services for fistula treatment, which is in line with previous studies [20, 47].

### Study strengths and limitations

The findings from this study add to the existing knowledge about experiences of living with obstetric fistula. The methods used may be transferable to respondents and settings similar to those of our study setting. The limitation of our study is that it excluded women from other regions of the country due to geographical inaccessibility as well as time and financial constraints; thereby limiting the perspectives, which might arise from differences in ethnicity; especially for the Northern region. However, similarities of our findings to other previous studies may also mean that ethnicity may not play a role in experiences of living with obstetric fistula [18, 19]. It was beyond the scope of our study to confirm or refute what participants reported about their spouses’ reactions to obstetric fistula, for example abandonment, divorce or reasons for remaining in the marriage. Therefore, future research might look into this area within the Malawian context. Other areas for future research may include effectiveness of sensitization of women and communities about complications during pregnancy, delivery and postnatal period and health seeking behaviors; traditional birth attendants′ roles in preventing obstetric fistula, repair and reintegration.

## Conclusion

Stigmatization still remains a major problem among women suffering from obstetric fistula in Malawi. The anticipation of stigma by women in this study consequently limited their social lives. We have argued that this fear of stigma might have arisen from previous knowledge of social norms concerning bowel and bladder control, which do not take into account an illness like fistula. This disregard might have also arisen from lack of knowledge about causes of fistula by both the affected women and their immediate family members. There is need therefore to create awareness and educate women and their communities about the causes of obstetric fistula, its prevention and treatment. This may help to prevent fistula as well as reduce all dimensions of stigma, and consequently increase dignity and quality of life for women with fistula.

## Abbreviations

HIV: Human Immunodeficiency Virus
MDHS: Malawi Demographic Health Survey
RVF: Rectovaginal Fistula
UNFPA: United Nations Population Fund
USA: United States of America
VVF: Vesicovaginal Fistula
WHO: World Health Organization
